# Acute Effects of Varying Neuromuscular Electrical Stimulation Amplitude on Quadriceps Isometric Torque and Muscle Thickness in Healthy Young Adults: A Randomized Split‐Limb Trial

**DOI:** 10.1002/pri.70300

**Published:** 2026-08-02

**Authors:** Jhonatan Zini dos Anjos, Otávio Augusto Correa Cenci, Isadora Giacomolli da Silva, Carlos Eduardo de Albuquerque, Gladson Ricardo Flor Bertolini, Alberito Rodrigo de Carvalho

**Affiliations:** ^1^ Universidade Estadual do Oeste do Paraná—UNIOESTE Western Paraná State University Cascavel Paraná Brazil

**Keywords:** electrical stimulation therapy, muscle strength, physical therapy modalities, quadriceps muscle

## Abstract

**Introduction:**

Despite the relevance of neuromuscular electrical stimulation (NMES) dosimetry, it is unclear whether increasing the self‐reported tolerated amplitude enhances acute neuromuscular responses.

**Objective:**

To investigate the acute effects of varying NMES amplitude and type of induced contraction on quadriceps isometric torque (IT) and muscle thickness.

**Methods:**

A randomized split‐limb within‐subject repeated‐measures design was employed in healthy young adults. IT and quadriceps muscle thickness were the outcomes. Bilateral measurements were obtained using participants seated at 60° (± 5°) of knee flexion. Muscle thickness was assessed via ultrasound and IT using a portable dynamometer. The Aussie current was applied at two doses: the self‐reported tolerated amplitude (threshold) and 20% above this value (suprathreshold). Doses were randomly applied to dominant and non‐dominant limbs across three assessment conditions in separate sessions: (1) IT at maximal voluntary effort without NMES, (2) IT during NMES alone, and (3) IT during NMES combined with maximal voluntary effort.

**Results:**

Forty‐one volunteers of both sexes participated. Isolated NMES (threshold = 0.49 and suprathreshold = 0.66 N m kg^−1^) elicited significantly lower IT than other conditions, whereas maximal voluntary IT (threshold = 2.82 and suprathreshold = 2.84 N m kg^−1^) did not differ from maximal voluntary IT performed concurrently with NMES (threshold = 3.03 and suprathreshold = 2.91 N m kg^−1^). The suprathreshold condition resulted in a greater increase in RF muscle thickness. Exploratory regression analysis showed that RF thickness was the only variable significantly associated with IT (*R* ^2^ = 0.54).

**Conclusions:**

Acute Aussie NMES did not increase IT compared with maximal voluntary contraction, and isolated NMES elicited lower IT. However, suprathreshold stimulation acutely increased RF thickness.

## 1 Introduction

Muscle strength is the ability to generate force against resistance, essential for musculoskeletal performance (Suchomel et al. 2016). Maximum strength reflects the neuromuscular system's capacity to produce peak force during voluntary contraction (Perrotta et al. 2019; Schoenfeld et al. 2021). Clinically, it is measured as maximum joint torque, calculated as muscle force multiplied by the moment arm, defined as the perpendicular distance between the force line of action and the joint's axis of rotation (Cavalcante et al. 2024; Risberg et al. 2018).

Given the significance of muscle strength in determining functionality, therapeutic interventions that augment muscle capacity to generate force hold considerable interest for professionals engaged in physical rehabilitation (Fyfe et al. 2022). Neuromuscular electrical stimulation (NMES) is a therapeutic modality that involves applying intermittent electrical stimulation to superficial skeletal muscles. The primary objective of NMES is to induce acute muscle contractions by activating motor nerve fibers, with the aim of eliciting neuromuscular chronic adaptations through repeated, systematic stimulation. NMES is widely used in clinical practice, with primary applications in functional recovery and, notably, in promoting long‐term neuromuscular gains (de Andrade et al. 2024; Nussbaum et al. 2017; Thomé et al. 2021).

In the acute phase, NMES promotes neural responses by depolarizing motor and sensory nerve fibers, potentially contributing to subsequent neural adaptations. As an immediate response in the acute phase, the stimulus directly depolarizes motor neurons under the electrode, reaching the spinal cord, where it activates sensory axons and reflexively recruits motor neurons, potentially enhancing strength gains during training (Collins 2007). Superimposing NMES onto voluntary muscle contractions may enhance muscle performance by simultaneously facilitating spinal and cortical excitability, as NMES acutely increases motor unit firing rates at higher force levels (Borzuola et al. 2025).

Effective muscle contraction requires precise NMES dosimetry, including amplitude, pulse duration, and electrode size. The appropriate combination of these parameters optimizes motor unit recruitment, promoting efficient contractions that acutely disrupt muscle homeostasis—characterized by metabolic and ionic perturbations and increased enzymatic activity—through externally induced contractions and non‐physiological recruitment patterns, thereby contributing to neuromuscular adaptations (Conley et al. 2021; Donnelly et al. 2021; Pereira et al. 2022). Current amplitude represents a challenging parameter to control during NMES dosing, as higher amplitudes are acutely associated with greater force output, motor unit recruitment, and quadriceps activation, but may concurrently elicit discomfort and accelerate fatigue (Glaviano and Saliba 2016).

There is still a gap in the literature regarding optimal NMES dosimetry for muscle strengthening, particularly whether higher current amplitudes increase mechanical stress and modulate motor unit recruitment, thereby influencing isometric torque (IT). This stems from the limited reporting of parameters and considerable heterogeneity when they are described (Pereira et al. 2022). It remains unclear whether tolerance‐based dosing, commonly used in practice, is sufficient to induce acute strength gains indicative of disrupted homeostasis or whether combining NMES with voluntary contraction yields greater strength than voluntary effort alone.

The objective of this study was to investigate the acute effects of varying NMES amplitude and type of induced contraction on quadriceps IT and muscle thickness. The hypothesis posited that suprathreshold doses—defined as doses greater than the self‐reported tolerated amplitude (threshold dose)— particularly when combined with maximal voluntary effort, would elicit greater IT and increased muscle thickness during NMES application.

## 2 Methods

### 2.1 Experimental Design and Ethics

This study employed a randomized within‐subjects repeated‐measures design with a split‐limb dose allocation approach. Because each participant received both stimulation conditions, no participant‐level randomization was performed. It was approved by the Western Paraná State University (Unioeste) ethics committee (Protocol No. 6.679.894) and registered in the Brazilian Clinical Trials Registry (RBR‐7ybw4gk). Eligible participants were informed about the study and provided written informed consent. We used DeepL, an AI‐based software, to review the grammar and style of the English writing.

### 2.2 Participants

Participants were recruited consecutively via digital media and in‐person approaches in a university neighborhood. To minimize the influence of age‐ and disease‐related factors, the sample comprised healthy young adults aged 18–27 years, both physically active and inactive. Exclusion criteria included neurological or cognitive disorders, cardiorespiratory conditions, ongoing musculoskeletal pain, recent musculoskeletal injuries, or a history of lower‐limb surgery. Because the study investigated the immediate effects of NMES in asymptomatic volunteers over three experimental sessions, therapeutic cointerventions were not considered relevant to the study design and were therefore not monitored.

### 2.3 Procedures

Data collection was conducted at the Laboratory of Physical Rehabilitation, Performance, and Integrative Biodynamics (BioRehab Lab) at Unioeste, Cascavel campus, Brazil. Recruitment and all measurements were conducted between June 2024 and March 2025.

The entire research team (three physiotherapy undergraduate students) was trained and calibrated prior to data collection by an experienced researcher who had previously published studies employing the same methodological procedures for these outcomes (Jakovacz et al. 2024). Pilot sessions were conducted over a 3‐month period before the start of the study.

**FIGURE 1:**
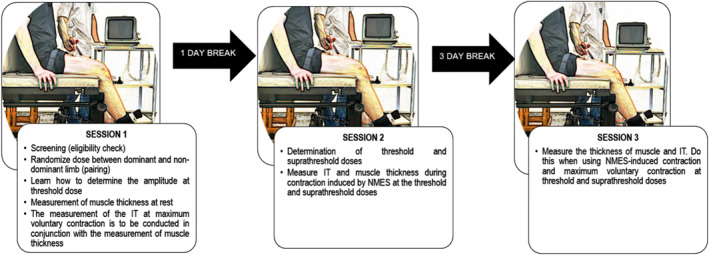
Schematic setup of data collection, representing the times of collection (sessions) and the procedures performed in each session. Isometric torque (IT) and neuromuscular electrical stimulation (NMES). *Source:* authors' own work.

Participants were individually instructed to attend the laboratory for three separate sessions, as illustrated in the accompanying diagram (Figure 1). On average, each session lasted between 60 and 90 minutes.

Outcome assessment was not blinded because the investigators responsible for NMES administration also performed all outcome assessments, including muscle thickness and torque measurements; therefore, assessors were aware of the stimulation condition during data collection. However, each researcher maintained the same role throughout the study to ensure procedural consistency. Given that maximal voluntary contraction typically recovers rapidly following fatigue‐induced task failure (Froyd et al. 2018), the minimum 24‐h interval between sessions was considered sufficient to minimize potential carryover effects.

The first session included eligibility screening and the collection of demographic and anthropometric data (sex, body mass, height, and limb dominance). For sample characterization purposes, physical activity was assessed using the short IPAQ, which is based on estimated energy expenditure. Estimated energy expenditure was calculated in MET‐min/week according to standard procedures (Craig et al. 2003). Participants were categorized as low (< 600 MET‐min/week), moderate (600–2999 MET‐min/week), or high (≥ 3000 MET‐min/week) based on the IPAQ scoring protocol. The validity of the IPAQ in the Brazilian context has been previously established (Matsudo et al. 2012; Vespasiano et al. 2012).

Before the intervention, an independent researcher not involved in data collection generated a computer‐based randomization sequence using GraphPad QuickCalcs. Allocation was performed at the limb level, with threshold (maximum subjectively tolerated amplitude) and suprathreshold NMES doses (+20% above threshold) randomly assigned to the dominant or non‐dominant limb. Given the nature of the intervention, in which both conditions involved active NMES delivered at perceptibly different stimulation intensities, participant blinding was not feasible. The dominant lower limb was defined as the limb preferentially used for kicking.

During familiarization, the current amplitude was increased until the participant reported intolerance. Subsequently, without NMES, the thickness of the rectus femoris (RF), vastus lateralis (VL), and vastus medialis (VM) was measured at rest, followed by bilateral assessment of knee extension IT and quadriceps thickness during maximum voluntary effort.

The next day, the volunteer returned to the laboratory for session 2. In this session, the amplitudes corresponding to the threshold and suprathreshold doses were determined. Based on the randomization established in the previous session, muscle thickness and IT were measured during NMES‐induced contractions at their respective doses, with no voluntary effort.

After a 3‐day interval, muscle thickness and IT were measured again using the same procedures as in session 2, except that the NMES‐induced contraction was augmented by maximal voluntary effort.

### 2.4 Evaluation of Outcomes

The measurements of quadriceps thickness and IT were performed using the knee flexed at an angle of 60° (with a tolerated variation of ± 5°). The choice of this angle was based on the angle‐torque relationship. This is because this angle yields the highest torque output (Eckert et al. 2024; Scott et al. 2021).

The thickness of the quadriceps muscle was measured using a portable ultrasound scanner (Shimadzu SDU450xl, Columbia, USA), equipped with a 7.5 MHz linear transducer. The identification of muscle analysis points was facilitated by measuring the distance from the anterior superior iliac spine to the upper edge of the patella using an anthropometric tape measure. The thigh circumference was measured at the midpoint of this distance (50%).

To obtain ultrasound images, the transducer was positioned transversely, perpendicular to the muscle fibers. Contact gel was meticulously applied between the transducer and the participant's skin, maintaining minimal pressure to avoid muscle compression. The location of the RF muscle was determined by placing the transducer at the midpoint (50% of the distance between the upper edge of the patella and the anterior superior iliac spine). In the case of the VL, the same 50% measurement was used, but the transducer was shifted laterally by 10% of the thigh circumference. To facilitate visualization of the VM, the transducer was positioned at 20% of this distance and shifted medially to 12.5% of the femoral circumference. This standardization in transducer placement aimed to ensure the reproducibility and accuracy of the measurements obtained, and followed the same parameters as a previous study (Jakovacz et al. 2024). Two ultrasound images of the muscle at rest were obtained, two during the maximum voluntary isometric contraction test, two during NMES, and two during the combination of NMES and maximum voluntary isometric contraction.

A portable dynamometer equipped with an inertial sensor (Dinabang‐Movi, Montevideo, Uruguay) was used to measure torque. The volunteer was seated on a custom‐adapted stretcher designed to facilitate muscle strength measurement (PhysioLab One, Cascavel, Brazil). The Dinabang was positioned on the participant's ankle brace and secured to the stretcher's structure with an inextensible chain. To minimize compensatory movements during the experimental trials, a strap was placed across the volunteer's thighs.

**FIGURE 2:**
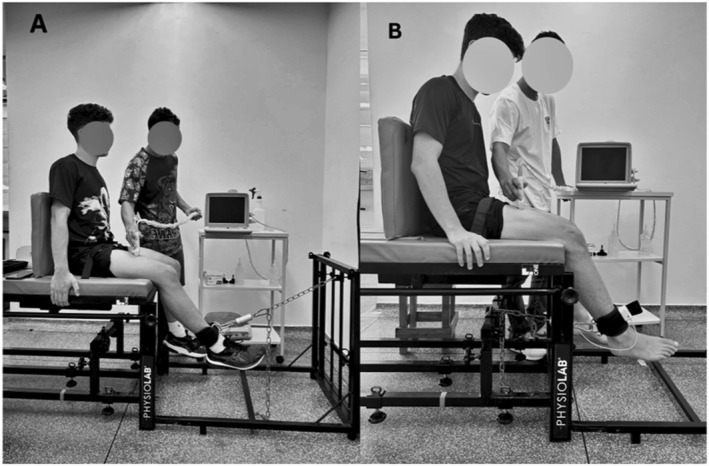
Schematic representation of quadriceps thickness measurements at rest (A) and during the maximum isometric torque test (B) with the knee at a 60° angle (with a tolerated variation of ± 5°) in flexion. *Source:* authors' own work.

Quadriceps thickness at rest was assessed with the limb immobilized by an inextensible chain, keeping the knee flexed at ∼60° and monitored by an inertial sensor (Figure 2A). For torque measurements, the same position was maintained, using a dynamometer applied to resist knee extension (Figure 2B).

For torque assessment, participants performed five‐second maximal knee extension efforts, each followed by 60 seconds of rest. Each limb completed six maximal contractions and six muscle thickness measurements (two for each muscle: RF, VM, and VL). The mean of these measurements was used for the statistical analysis of each outcome. Perceived exertion was assessed using the Borg scale (Borg 1982).

### 2.5 Electrostimulation Protocol

**FIGURE 3:**
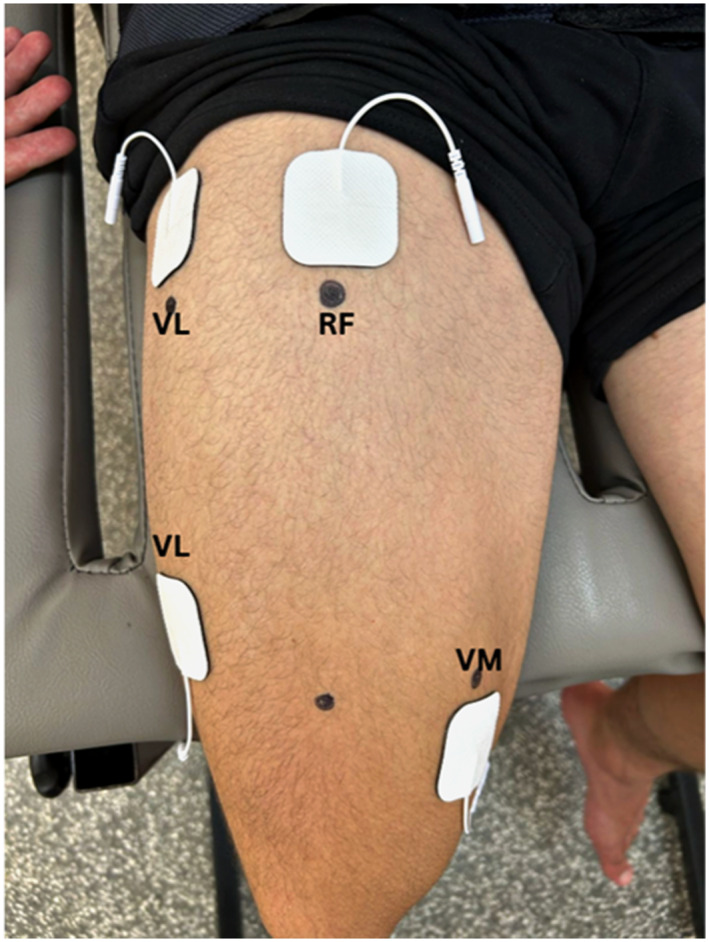
Positioning of electrodes at the motor point of each muscle, image for illustrative purposes only, as the motor point may differ in each individual. RF, rectus femoris; VL, vastus lateralis; VM, vastus medialis. *Source:* authors' own work.

An electrostimulation device (Neurodyn Esthetic, Ibramed, Amparo, Brazil) was used to deliver NMES using Aussie current. Stimulation parameters included a carrier frequency of 1 kHz, a burst duration of 2 ms, an “ON” time of 7 s, and an “OFF” time of 60 s. Two stimulation channels were used, with 4 × 4 cm self‐adhesive electrodes positioned over the vastus lateralis (VL) for one channel and over the rectus femoris (RF) and vastus medialis (VM) for the other (see Figure 3).

Stimulation amplitude was individually adjusted for each channel. The threshold dose was defined as the maximum subjectively tolerated amplitude achieved during each session, determined by progressively increasing the current until the participant reported intolerance. This procedure was repeated at the beginning of each session to account for day‐to‐day variability in tolerance.

The suprathreshold dose was defined as 20% above the threshold amplitude determined within the same session, ensuring that the relative increase was individualized. The +20% increment was selected as a pragmatic and exploratory approach to provide a stimulus clearly above the tolerated threshold while maintaining safety and feasibility, given the absence of standardized dose–response guidelines for NMES amplitude (Nussbaum et al. 2017).

When two channels were used, the stimulation “dose” was operationalized as the relative increase in amplitude (threshold vs. suprathreshold) applied independently to each channel, rather than as a summed or combined output. The motor point was identified by delivering low‐intensity pulses to the quadriceps (RF, VM, VL) and gradually increasing the current amplitude until a visible muscle twitch was observed. The location eliciting the strongest response was marked with a dermographic pen to guide electrode placement. Intervention details are described according to the TIDieR checklist, which is provided as supplementary material (see TIDieR checklist in the supplementary material).

### 2.6 Data Processing

The thickness of each part of the quadriceps femoris muscle was measured from the ultrasound images using ImageJ software (National Institute of Health, Bethesda, MD, USA, version 1.49). Initially, the images were calibrated by measuring the number of pixels that corresponded to a known distance of 39 mm.

**FIGURE 4:**
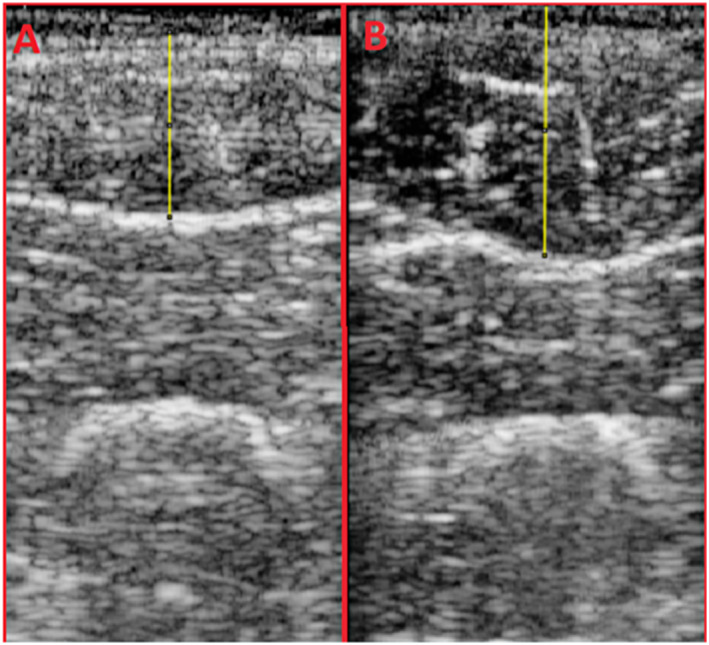
Example of a measurement of the thickness of the rectus femoris at rest (A) and maximum voluntary contraction (B). *Source:* authors' own work.

The thickness was determined by measuring the distance between the superficial fascia of the muscle and the femur for the VM, as well as the distance between the superficial fascia and the deep fascia of the muscle in the direction of the femur's most superficial point for the RF and VL. The final value of muscle thickness at rest and during the strength test was obtained by averaging the two images for each muscle. An illustration of an RF measurement, both at rest and during contraction, is presented in Figure 4.

For torque measurement, the lever arm was defined as the distance from the knee center to the chain attachment on the anklet, and this value was entered into the Dinabang application. Torque was automatically computed in the Dinabang app by multiplying the measured force (N) by the lever arm length (m). The obtained torque values were normalized to body mass (N m kg^−1^).

### 2.7 Statistical Analysis

The primary outcome was IT. Muscle thickness was analyzed as a secondary outcome. The main comparisons of interest were between NMES doses (threshold vs. suprathreshold) and contraction conditions (NMES alone, maximal voluntary effort, and NMES combined with maximal voluntary effort). The sample size was calculated using IT values from Eckert and colleagues (Eckert et al. 2024), with effect size = 0.35, *α* = 0.05, power = 0.90, three groups, and two doses, resulting in a minimum of 20 volunteers (G*Power 3.1.9.7, ANOVA: repeated measures, within‐between interaction). Data were analyzed in Jamovi 2.7.9 with significance set at 5% (*α* = 0.05).

To account for repeated measures and within‐subject correlations, regression‐based models were used. The choice of model was guided by the distributional characteristics of each outcome. Initially, both Gaussian (linear) and gamma (non‐Gaussian) models were fitted. Model selection was based on residual diagnostics and the Akaike Information Criterion (AIC), with the model showing the best fit (lowest AIC) retained.

When the data were adequately modeled with a Gaussian distribution, Generalized Estimating Equations (GEE) were applied, given their robustness to correlated data under the assumption of normality. When the gamma distribution provided a better fit, a Generalized Mixed Model (GMM) was used to account for non‐normality and positive skewness in the data. Depending on the selected model, statistical tests are reported as F‐statistics (for GEE models) or *χ* ^2^‐statistics (for GMM), reflecting differences in the underlying estimation and inference frameworks.

All models tested the main effects of dose and contraction type, as well as their interaction. For muscle thickness, stimulation amplitude was included as a covariate. Where appropriate, results are presented as estimated marginal means with corresponding confidence intervals, in addition to *p*‐values, to facilitate interpretation of effect magnitude.

The association between IT and muscle thickness during maximal voluntary contraction was analyzed using a linear regression model and is reported as an exploratory analysis, without implying predictive sufficiency. Reproducibility was assessed using the intraclass correlation coefficient (ICC [3, k]) and the standard error of measurement (SEM) for IT and muscle thickness measurements.

Additional analyses, including resting muscle thickness, secondary outcomes, and detailed model selection procedures, are reported in the supplementary material (see Supporting Information S1, Tables S1–S4).

## 3 Results

### 3.1 Participant Characteristics

A total of 75 individuals expressed interest in participating in the study, but 34 were excluded because of lower‐limb injuries, pain, or a history of lower‐limb surgery. Of the remaining individuals, 41 volunteers were enrolled, and all completed the three scheduled experimental sessions (100% adherence), with no losses to follow‐up, protocol deviations, or intervention discontinuations. All participants successfully reached the predefined threshold and suprathreshold stimulation amplitudes, and no protocol adjustments were required due to intolerance. Based on the mean estimated energy expenditure (MET‐min/week), the sample was characterized as having a moderate level of physical activity. All participants reached the predefined stimulation amplitudes, including the suprathreshold condition. No participants required protocol adjustment or were excluded due to intolerance.

Descriptive statistics for the sample characteristics are presented in Table 1.

**TABLE 1:** Descriptive statistics for sample characterization variables.

Variable	*n*	Mean	(±) standard deviation	95% confidence interval	
				Lower limit	Upper limit
Age (years)	41	20.1	2.07	19.4	20.8
Body mass (kg)	41	70.4	19.2	64.3	76.4
Height (m)	41	1.71	0.08	1.68	1.73
BMI (kg·m^−2^)	41	23.9	5.1	22.3	25.5
Estimated energy expenditure (MET‐min/week)	41	2514	99	2389	2640
Sex	♂ = 20				
	♀ = 21				
Limb dominance	Right = 37				
	Left = 4				

Abbreviations: BMI, Body Mass Index; MET, Metabolic Equivalent.

On average, the current amplitude at the threshold dose was 51.2 ± 14.6 mA for RF + VM and 58.0 ± 17.4 mA for VL, whereas at the suprathreshold dose, they were 59.3 ± 16.7 mA for RF + VM and 64.7 ± 18.7 mA for VL. Current amplitude was significantly higher in the suprathreshold than in the threshold dose for both RF + VM (F[1; 200] = 48.8, *p* < 0.001) and VL (F[1; 200] = 28.7, *p* < 0.001).

Perceived exertion, as measured by the Borg scale, differed across contraction conditions, being lowest during NMES alone, higher during maximal voluntary effort, and highest when both were combined, irrespective of dose (statistical details are provided in the “Borg scores” section of the supplementary material).

The linear regression yielded an *R* ^2^ = 0.54, and RF thickness was the only variable significantly associated with IT (RF: *t* = 5.041, *p* < 0.001; VM: *t* = 0.733, *p* = 0.466; VL: *t* = 1.377, *p* = 0.173).

IT was influenced by contraction type (*F* [2; 190] = 575.00, *p* < 0.01), but not for dose (*F* [1; 188] = 0.066, *p* = 0.797) and dose*contraction interaction (*F* [2; 188] = 1.870, *p* = 0.157).

RF thickness was influenced by dose (*χ* ^2^[1] = 3.973, *p* = 0.046) and contraction type (*χ* ^2^[2] = 147.4, *p* < 0.001), but it was not affected by channel amplitude (*χ* ^2^[1] = 3.881, *p* = 0.348) or the dose*contraction interaction (*χ* ^2^[2] = 1.975, *p* = 0.372). VM thickness was influenced only by contraction type (*F* [2; 194] = 20.66, *p* < 0.001), with no effect of dose (*F* [1; 199] = 0.481, *p* = 0.489), channel amplitude (*F* [1; 217] = 0.0005, *p* = 0.982), or the dose*contraction interaction (*F* [2; 195] = 0.485, *p* = 0.617). VL thickness was not affected by any factor—dose (*χ* ^2^[1] = 0.103, *p* = 0.748), contraction type (*χ* ^2^[2] = 3.901, *p* = 0.142), channel amplitude (*χ* ^2^[1] = 1.591, *p* = 0.207), or the dose*contraction interaction (*χ* ^2^[2] = 0.934, *p* = 0.627).

**FIGURE 5:**
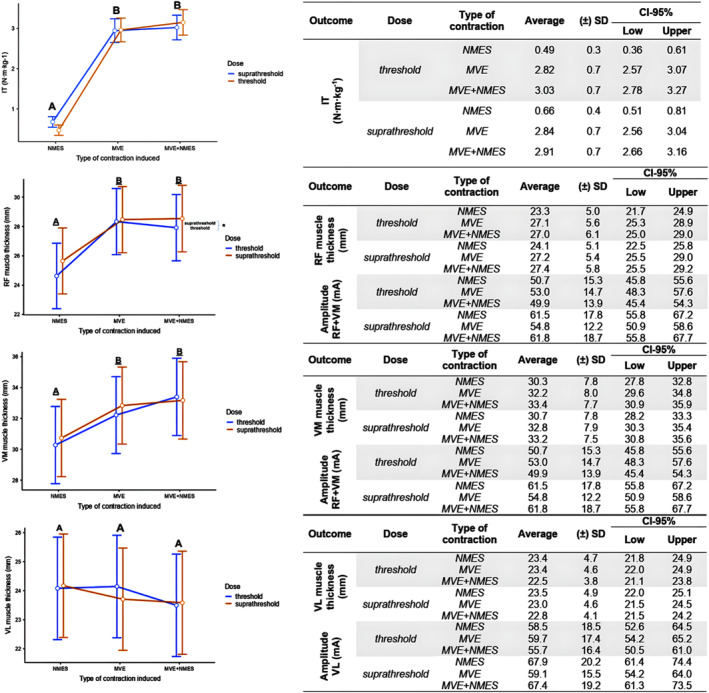
Descriptive and inferential statistics for isometric torque (IT) and muscle thickness (RF, VM, VL) across dose and contraction conditions. The accompanying table presents mean (SD) values and 95% confidence intervals (CI) for all outcomes. Amplitude was modeled as a covariate in thickness analyses. Isometric torque (IT), maximum voluntary effort (MVE), neuromuscular electrical stimulation (NMES), rectus femoris (RF), vastus lateralis (VL), and vastus medialis (VM). Different letters indicate statistical differences among contraction conditions, and asterisks indicate statistical differences between doses.

For RF, the suprathreshold dose increased RF thickness by 0.59 mm (95% CI [0.009 to 1.18]) compared with the threshold dose, corresponding to approximately a 1.94% increase. For both RF and VM, NMES alone produced smaller changes than the other contraction conditions. The descriptive and inferential statistics for the effects of the factors on all outcomes are shown in Figure 5.

Reproducibility metrics for muscle thickness and torque measurements are presented in Tables 2 and 3. Overall, ICC values ranged from 0.981 to 0.994, indicating good‐to‐excellent reliability, whereas SEM values ranged from 0.703 to 1.006.

**TABLE 2:** Reproducibility of outcome measures regardless of muscle thickness.

	MVE			NMES			MVE + NMES		
	RF	VL	VM	RF	VL	VM	RF	VL	VM
ICC (%)	0.989	0.987	0.992	0.981	0.982	0.992	0.993	0.983	0.994
95% CI (lower to upper)	0.984 to 0.992	0.981 to 0.991	0.989 to 0.995	0.972 to 0.987	0.974 to 0.987	0.988 to 0.994	0.990 to 0.995	0.976 to 0.988	0.992 to 0.996
Strength	Excellent	Excellent	Excellent	Excellent	Excellent	Excellent	Excellent	Excellent	Excellent
CV (%)	3.25	3.14	3.06	3.98	3.93	3.29	2.56	3.23	2.41
SEM (mm)	0.703	0.704	0.946	0.941	0.922	1.006	0.698	0.733	0.801

Abbreviations: 95% CI, 95% confidence interval; CV, coefficient of variation; ICC, intraclass correlation coefficients; MVE, maximum voluntary effort; NMES, neuromuscular electrical stimulation; RF, rectus femoris; SEM, standard error of measurement; VL, vastus lateralis; VM, vastus medialis.

**TABLE 3:** Reproducibility of outcome measures regardless of isometric torque.

	IT		
	MVE	NMES	MVE + NMES
ICC (%)	0.993	0.974	0.992
95% CI (lower to upper)	0.990 to 0.995	0.961 to 0.982	0.989 to 0.995
Strength	Excellent	Excellent	Excellent
CV (%)	4.32	18.04	4.41
SEM (N.m)	0.870	0.665	0.919

Abbreviations: 95% CI, 95% confidence interval; CV, coefficient of variation; ICC, intraclass correlation coefficients; IT, isometric torque; MVE, Note: maximum voluntary effort; NMES, neuromuscular electrical stimulation; SEM, standard error of measurement.

## 4 Discussion

The study aimed to examine the immediate effects of different NMES amplitude doses and contraction types on IT and quadriceps muscle thickness. The hypothesis was partially refuted, as suprathreshold stimulation affected only RF thickness. Borg scores increased across contraction conditions but were not affected by NMES dose (statistical details are provided in the “Borg scores” section of the supplementary material).

The Borg scale primarily reflects perceived exertion rather than stimulation‐related discomfort. Accordingly, the lower Borg scores observed during NMES alone likely reflect the reduced voluntary effort required in this condition compared with maximal voluntary contractions. Although NMES may induce sensory discomfort, this construct was not directly assessed. Therefore, the present findings should be interpreted as differences in perceived exertion rather than differences in discomfort or pain.

Kramer (Kramer 1987) compared isometric knee extension torque during maximum voluntary contraction with electrical stimulation. Torque from electrostimulation was lower than voluntary effort, and the combination offered no additional gain. The current study supports these findings, showing no additional benefit of NMES combined with voluntary contraction on IT. Importantly, in healthy individuals, acute NMES—either alone or superimposed—does not augment torque beyond maximal voluntary contraction, suggesting limited additive benefit of electrically evoked activation over voluntary drive. Practically, NMES alone does not appear to enhance acute torque output, and these findings should not be extrapolated to long‐term adaptations.

Although NMES is known to promote chronic adaptations such as torque gains and hypertrophy (Cittadin et al. 2020; Gondin et al. 2011), the acute increase in RF thickness observed with suprathreshold dosing in the present study should be interpreted with caution. Whether such transient responses contribute to long‐term adaptations with repeated exposure remains to be determined.

The relationship between IT and RF can be explained by RF's role as the primary muscle in knee extension and torque generation during isometric contractions. Evidence shows a direct link between RF activation and torque (Pethick et al. 2019). Torque magnitude is influenced by muscle thickness and, more importantly, by neural activation and motor unit recruitment (Lattier et al. 2004; Enoka and Duchateau 2016). Thus, while RF thickness contributes to torque, neural efficiency is the dominant factor. This helps explain why torque often increases before hypertrophy, suggesting improved coordination and recruitment as underlying mechanisms (Del Vecchio et al. 2019). Indeed, the variation in RF thickness observed in the present study accounts for only 54% of IT, supporting the notion that increases in IT are not solely dependent on muscle thickness.

The increase in muscle strength from high‐intensity stress, including NMES, arises from both neurological and morphological mechanisms. The main factor is greater motor unit recruitment and improved synchronization of contractions (Aagaard et al. 2002). Strength training also induces central nervous system adaptations that enhance activation efficiency, leading to significant gains in muscle strength even before visible hypertrophy (Nummela et al. 2006). Thus, torque improvements are largely attributable to enhanced neural activation, a key factor in NMES‐induced strength gains. However, based on our findings, achieving such gains likely requires repeated exposure over time, as these adaptations are expected to occur only with repeated application of the stimulus.

This study has some limitations. The use of a healthy young sample limits generalizability to clinical populations. In addition, discomfort or pain associated with NMES was not systematically quantified. Because perceptual responses may influence motor output, tolerance, and task performance during electrically evoked contractions, the absence of these measures limits the interpretation of dose‐response effects, particularly regarding the lack of additional torque production under suprathreshold stimulation. Although familiarization was performed, potential learning or motivational effects across sessions cannot be ruled out. Finally, as only acute responses were evaluated, the findings cannot be extrapolated to long‐term adaptations.

Participant blinding was not feasible because both experimental conditions involved active NMES delivered at perceptibly different stimulation intensities. Although this may have influenced subjective secondary outcomes, such as perceived exertion, the primary outcome (IT) was measured objectively using dynamometry, thereby reducing the potential for expectation bias on the main findings.

The selected current is known to generate high torques and is generally well tolerated (Ward 2009; Ward and Lucas‐Toumbourou 2007; Ward and Robertson 1998). Future studies should investigate alternative electrostimulation strategies and systematically assess perceptual responses alongside neuromuscular outcomes. Additional research is also needed to evaluate NMES effects on torque, considering variables such as exposure time, intensity, frequency, and long‐term adaptations, to better clarify its clinical relevance and optimize its use in rehabilitation. In contrast, in clinical populations characterized by impaired voluntary activation, such as individuals with arthrogenic muscle inhibition following knee injury or surgery, NMES may play a more relevant role in restoring muscle activation and improving torque.

## 5 Conclusion

Acute NMES with an Aussie current did not further increase IT beyond maximal voluntary contraction. However, suprathreshold stimulation was associated with an acute increase in RF thickness, but not with a corresponding increase in IT.

## 6 Implications of Physiotherapy Practice

These findings may inform the development of NMES protocols and the design of future clinical trials. Although higher stimulation amplitudes appear feasible, the lack of acute torque enhancement and the potential for discomfort should be considered when defining dosing strategies. These effects may be particularly relevant in populations with impaired voluntary activation, such as individuals with quadriceps inhibition following knee surgery. However, the clinical efficacy of such approaches remains to be established and should be investigated in these populations.

## Funding

The first and second authors received scholarships for a university outreach project supported by the Fundação Araucária and the Unioeste.

## Ethics Statement

This study was approved by the institutional ethics committee (Protocol No. 6.679.894) and registered in the Brazilian Clinical Trials Registry (RBR‐7ybw4gk).

## Conflicts of Interest

The authors declare no conflicts of interest.

## Supporting information


Supporting Information S1

Supporting Information S2

## Data Availability

Research data are not shared.
