# Supplementary material for: Acute Effects of Varying Neuromuscular Electrical Stimulation Amplitude on Quadriceps Isometric Torque and Muscle Thickness in Healthy Young Adults: A Randomized Split‐Limb Trial
Source: Physiother Res Int. 2026 Aug 2;31(4):e70300. doi: 10.1002/pri.70300 (PMC13429896; doi:10.1002/pri.70300)
Supplement: Supplementary file 1 — Supporting Information S1 [file PRI-31-e70300-s002.docx]

**SUPPLEMENTARY MATERIAL**

**Comparisons of muscle thickness between the resting condition and each of the other conditions.**

Model fit was assessed using linear (normal) and gamma (non-normal) distributions, adopting an identity link function for both. The model with the lowest Akaike Information Criterion (AIC) was considered the best fit. Comparisons were first made between muscle thickness at rest and under the other experimental conditions. Table S1 summarizes the corresponding AIC values, and Table S2 shows the descriptive statistics for muscle thickness across all conditions.

**Table S1:** Akaike Information Criterion (AIC) values from the comparisons of muscle thickness at rest with the other conditions.

|  | rectus femoris (RF) | vastus lateralis (VL) | vastus medialis (VM) |
| --- | --- | --- | --- |
| **LINEAR** | **1550** | **1525** | **1730** |
| **GAMMA** | **1476** | **1443** | **1718** |

**Note:** The bolded values indicate the distribution that provided the best fit to the data

**Table S2.** Descriptive statistics (means and standard deviations) for muscle thickness across all conditions—rest, maximum voluntary effort (MVE), neuromuscular electrical stimulation (NMES), and their combination (MVE + NMES)—separated by side of dominance.

|  | **Rest** | | **MVE** | | **NMES** | | **MVE+NMES** | |
| --- | --- | --- | --- | --- | --- | --- | --- | --- |
|  | **DOMINANT** | **NO DOMINANT** | **DOMINANT** | **NO DOMINANT** | **DOMINANT** | **NO DOMINANT** | **DOMINANT** | **NO DOMINANT** |
| **RF** | 21.6 ± 4.8 | 21.6 ± 4.6 | 27.0 ± 5.4 | 27.3 ± 5.6 | 23.7 ± 4.8 | 23.5 ± 4.8 | 27.2 ± 5.7 | 27.3 ± 5.9 |
| **VL** | 22.4 ± 4.1 | 22.5 ± 4.4 | 23.6 ± 4.5 | 22.8 ± 4.7 | 23.0 ± 4.7 | 23.9 ± 4.9 | 22.7 ± 4.1 | 22.7 ± 3.9 |
| **VM** | 31.4 ± 7.7 | 30.6 ± 7.4 | 32.5 ± 8.3 | 32.5 ± 7.6 | 30.7 ± 8.0 | 30.3 ± 7.6 | 33.4 ± 7.5 | 33.1 ± 7.8 |

**Legend:** maximum voluntary effort (MVE); neuromuscular electrical stimulation (NMES); rectus femoris (RF); vastus lateralis (VL); vastus medialis (VM).

There was no main effect of the side of dominance for any muscle (RF: χ²[1] = 0.0283, p = 0.866; VL: χ²[1] = 0.0482, p = 0.826; VM: χ²[1] = 0.0661, p = 0.797), nor was there an interaction effect between condition and side of dominance (RF: χ²[3] = 0.3916, p = 0.942; VL: χ²[3] = 8.225, p = 0.052; VM: χ²[3] = 1.6704, p = 0.644). However, there was a significant main effect of condition for all muscles (RF: χ²[3] = 425.12, p < 0.001; VL: χ²[3] = 10.621, p = 0.014; VM: χ²[3] = 50.023, p < 0.001). The pairwise differences among conditions are presented in Table S3. Overall, muscle thickness tended to be greater in the experimental conditions than at rest, with the largest increases observed during MVE. In contrast, NMES and MVE + NMES did not consistently elicit such increases.

**Table S3.** Differences in muscle thickness (mm) between rest and the other experimental conditions, along with their statistical significance (p-values). Positive values indicate greater muscle thickness compared to rest, whereas negative values indicate lower thickness relative to rest.

|  | **MVE - rest** | **NMES - rest** | **MVE+NMES - rest** |
| --- | --- | --- | --- |
| **RF** | 5.23 (p<0.001) | 1.92 (p<0.001) | 5.19 (p<0.001) |
| **VL** | 0.65 (p = 0.030) | 0.91 (p = 0.003) | 0.29 (p = 0.314) |
| **VM** | 1.66 (p<0.001) | -0.29 (p = 0.500) | 2.42 (p<0.001) |

**Legend:** maximum voluntary effort (MVE); neuromuscular electrical stimulation (NMES); and their combination (MVE + NMES); rectus femoris (RF); vastus lateralis (VL); vastus medialis (VM).

**Selection of the best-fit model for mean outcomes**

Model fit for the mean outcomes was assessed using linear (normal) and gamma (non-normal) distributions. The model with the lowest Akaike Information Criterion (AIC) was considered the best fit, and an identity link function was applied to both models. The corresponding results are presented in Table S4.

**Table S4:** Akaike Information Criterion (AIC) values from the comparisons of mean outcomes.

| **OUTCOME** | **LINEAR** | **GAMMA** |
| --- | --- | --- |
| Subjective perception of effort (Borg scale) | **1100** | the model did not converge for this outcome |
| Maximal isometric torque | 437 | **431** |
| Rectus femoris (RF) muscle thickness | 1216 | **1133** |
| Vastus medialis (VM) muscle thickness | **1340** | the model did not converge for this outcome |
| Valtus lateralis (VL) muscle thickness | 1185 | **1102** |

**Note:** Bolded values indicate the best-fit model for the dataset.

**Borg scores**

In the threshold dose, Borg scores were **2.3±2.8 points** during NMES alone**, 3.9±2.6 points** during maximal voluntary effort, and **5.0±3.1 points** during NMES combined with maximal voluntary effort. In the suprathreshold dose, the corresponding values were **2.7±3.1, 4.0±2.7**, and **5.1±3.0 points**, respectively. A main effect of **contraction type** was identified (F[2] = 36.692, p < 0.001), whereas neither **dose** (F[1] = 0.507, p = 0.477) nor the **dose*contraction interaction** (F[2] = 0.129, p = 0.879) influenced perceived exertion. On average, Borg scores were **1.4 points higher** during maximal voluntary effort without NMES and **2.5 points higher** during NMES combined with maximal voluntary effort, relative to NMES alone. The Borg score during NMES combined with maximal voluntary effort was, on average, 1.1 points higher than during maximal voluntary effort without NMES. Because the Borg scale assesses perceived exertion, these findings indicate greater perceived exertion under conditions involving voluntary contractions than with NMES alone.
