# Supplementary material for: Acute Effects of Varying Neuromuscular Electrical Stimulation Amplitude on Quadriceps Isometric Torque and Muscle Thickness in Healthy Young Adults: A Randomized Split‐Limb Trial
Source: Physiother Res Int. 2026 Aug 2;31(4):e70300. doi: 10.1002/pri.70300 (PMC13429896; doi:10.1002/pri.70300)
Supplement: Supplementary file 2 — Supporting Information S2 [file PRI-31-e70300-s001.docx]

| **Item**  **number** | **Item** | **Description** |
| --- | --- | --- |
| 1 | Brief name | Immediate effects of altering neuromuscular electrical stimulation (NMES) amplitude and contraction type on quadriceps muscle function. |
| 2 | Why | Neuromuscular electrical stimulation (NMES) is a therapeutic modality that involves the application of intermittent electrical stimuli to superficial skeletal muscles. The primary objective of NMES is to promote muscle contractions through the activation of muscle nerve fibers. The utilization of NMES in clinical practice has been extensive, with its primary applications being in the domain of functional recovery and, notably, enhancing muscle strength. NMES promotes neural adaptations by depolarizing motor and sensory branches. The stimulus directly depolarizes motor neurons under the electrode, reaching the spinal cord, where it activates sensory axons and reflexively recruits motor neurons, potentially enhancing strength gains during training. Superimposing NMES onto voluntary muscle contractions may enhance muscle performance by simultaneously facilitating spinal and cortical excitability, as NMES acutely increases motor unit firing rates at higher force levels.  There is still a gap in the literature regarding optimal NMES dosimetry for muscle strengthening. This stems from limited reporting of parameters and considerable heterogeneity when they are described. It remains unclear whether tolerance-based dosing, commonly used in practice, is sufficient to induce immediate strength gains indicative of disrupted homeostasis, or if combining NMES with voluntary contraction produces greater strength than voluntary effort alone |
| 3 | What (materials) | The thickness of the quadriceps muscle was measured using a portable ultrasound scanner (Shimadzu SDU450xl, Columbia, USA), equipped with a 7.5 MHz linear transducer. To obtain ultrasound images, the transducer was positioned transversely, perpendicular to the muscle fibers. Contact gel was meticulously applied between the transducer and the participant's skin, maintaining minimal pressure to avoid muscle compression. The thickness of each part of the quadriceps femoris muscle was measured from the ultrasound images using ImageJ software (National Institute of Health, Bethesda, MD, USA, version 1.49).  A portable dynamometer equipped with an inertial sensor (Dinabang - Movi, Montevideo, Uruguay) was utilized to measure torque. The volunteer was seated on a custom-adapted stretcher specifically designed to facilitate the measurement of muscle strength (PhysioLab One, Cascavel, Brazil). To mitigate the occurrence of compensatory movements during the experimental trials, a strap was strategically positioned across the volunteer's thighs.  The electrostimulation device (Neurodyn Esthetic Ibramed, Amparo, Brazil) was utilized to administer NMES through the use of Aussie current. The experimental protocol used two channels, with 4 × 4 cm self-adhesive electrodes placed on the VL muscle for one channel and on the RF and VM muscles for the other. |
| 4 | What (procedures) | The electrostimulation device was utilized to administer NMES through the use of Aussie current. The parameters employed in this study included a frequency of 1 kHz, a burst of 2 milliseconds, an “ON” time of 7 seconds, and an “OFF” time of 60 seconds. The experimental protocol used two channels, with 4 × 4 cm self-adhesive electrodes placed on the VL muscle for one channel and on the RF and VM muscles for the other. The volunteers took part in three separate sessions. At the first, the threshold (maximum subjectively tolerated amplitude) and suprathreshold over threshold amplitude (+20%) NMES doses were alternately assigned to the dominant and non-dominant limbs; additionally, the side assessed first was randomized to avoid order effects. The 20% suprathreshold increment was arbitrarily defined to provide a stimulation level distinctly higher than the threshold, yet still tolerable for participants. During familiarization, current amplitude was increased until the participant reported intolerance. At the second, the amplitudes corresponding to the threshold and suprathreshold doses were determined. Based on the randomization established in the previous session, muscle thickness and IT were measured during contractions induced solely by NMES at their respective doses, with no voluntary effort. At the third, muscle thickness and IT were measured again following the same procedures as in session 2, but this time the NMES-induced contraction was augmented by maximal voluntary effort. |
| 5 | Who provided | The entire research team (three physiotherapy undergraduate students) was trained and calibrated prior to data collection by an experienced researcher who had previously published studies employing the same methodological procedures for these outcomes. Pilot sessions were conducted over a three-month period before the start of the study. Blinding of outcome assessment was not feasible, as the investigators who administered NMES also served as outcome assessors; however, each researcher’s role was fixed throughout the study to ensure consistency in data collection. |
| 6 | How | Participants were individually instructed to attend the laboratory for three separate sessions. On average, each session lasted between 60 and 90 minutes. |
| 7 | Where | Participants were consecutively recruited through digital media and personal approaches surrounding of a university neighborhood. Data collection was conducted at the Laboratory of Physical Rehabilitation, Performance, and Integrative Biodynamics (BioRehab Lab) at Unioeste, Cascavel campus, Brazil. |
| 8 | When and how much | Recruitment and all measurements were conducted between June 2024 and March 2025. In each of the three sessions attended by the volunteers, NMES was administered as follows:  In the first session, participants underwent a familiarization procedure to understand how the threshold amplitude was determined and what the procedure and associated sensations would be like. During this session, torque and muscle thickness were measured during maximal voluntary contraction without the influence of electrical stimulation;  One day later, in the second session, after determining the threshold and suprathreshold doses, participants were assessed at both doses (each applied to one limb according to the randomization sequence). In this session, torque and muscle thickness were evaluated solely under NMES-induced contraction;  Three days later, the third session followed the same assessment procedures; however, in this stage, NMES was combined with maximal voluntary contraction. |
| 9 | Tailoring | All NMES parameters were identical across sessions, and the only parameter that was adjusted was the amplitude, as it depended on the participant’s self-perceived tolerance. The procedures used to assess the outcomes were identical across all sessions. |
| 10 | Modifications | No changes were necessary during the development of the study. |
| 11 | How well (planned) | The team was trained and calibrated until they felt fully prepared to begin data collection. The procedures were frequently supervised by the advising professor. The methodological pattern remained consistent throughout the entire study. |
| 12 | How well (actual) | All participants enrolled in the study completed all three sessions, with no losses to follow-up. |
